# What is available to support pain management in Parkinson’s: a scoping review protocol

**DOI:** 10.1186/s12939-023-02046-7

**Published:** 2023-11-23

**Authors:** Mark Parkinson, Cormac Ryan, Leah Avery, Annette Hand, Bhanu Ramaswamy, Julie Jones, Fiona Lindop, Monty Silverdale, Katherine Baker, Jenni Naisby

**Affiliations:** 1 Faculty of Health & Life Sciences, Department of Sport, Northumbria University, Exercise & Rehabilitation, Coach Lane Campus, Coach Lane, Newcastle-Upon-Tyne, UK; 2 Teesside University, Centre for Rehabilitation, Middlesbrough, Tees Valley UK; 3 Faculty of Health & Life Sciences, Department: Nursing, Northumbria University, Midwifery & Health, Coach Lane Campus, Coach Lane, Newcastle-Upon-Tyne, UK; 4 Newcastle Upon Tyne Hospitals NHS Foundation Trust, Newcastle-Upon-Tyne, UK; 5 Sheffield Hallam University, Sheffield, UK; 6 Robert Gordon University, School of Health Sciences, Garthdee Road, Aberdeen, UK; 7 University Hospitals of Derby & Burton NHS Foundation Trust, Derby, UK; 8 Department of Neurology, Manchester University, Manchester Centre for Clinical Neurosciences, Northern Care Alliance NHS Foundation Trust, Salford, UK

**Keywords:** Parkinson’s disease, Pain, Self-management, Behavioural interventions, Behaviour change

## Abstract

**Objective:**

A scoping review will be undertaken to examine and map the available evidence that has been produced in relation to pain management in Parkinson’s, with a focus on behavioural interventions, resources and/or how professionals support people with Parkinson’s self-management of pain.

**Methods:**

This review will be based on the methodological framework given by Arksey and O’Malley’s (2005), including enhancements by Levac et al., Peters et al. and the Joanna Briggs Institute. We will include studies from PubMed, SCOPUS, CINAHL, MEDLINE Web of Science, APA PsycINFO and ASSIA from January, 2010 onwards. Both quantitative and qualitative data will be analysed separately to identify the characteristics of support for pain management available, orientation of the approach and any identifiable behaviour change components and their outcomes. The COM-B behaviour change model and Theoretical Domains Framework will provide a theoretical framework for synthesising evidence in this review.

**Conclusion:**

This scoping review will help to explore studies focusing on the evidence supporting a range of interventions relating to the management of pain experienced by people living with Parkinson’s. The focus will be on describing what is available to support self-management, identify what behaviour change components have been used and their effectiveness, identify barriers and enablers to pain management and explore gaps in current provision of pain management. This review will identify implications and priorities for the follow-up phases to the larger ‘Pain in Parkinson’s’ Project which is designed to support clinicians and individuals living with Parkinson’s.

## Introduction

The operational definition of ‘people living with Parkinson’s (PwP) adopted in this scoping review includes people with a diagnosis of Parkinson’s disease (PD) according to the U.K. Brain Bank Criteria. The review is not aimed towards people who demonstrate features indicating another type of degenerative parkinsonism (also known as atypical Parkinson’s disease or Parkinson’s plus), e.g., progressive supranuclear palsy. Parkinsonism may produce symptoms, such as movement problems which mimic those seen in Parkinson’s disease, including tremors, slow movement and stiffness. However, parkinsonism tends to progress more rapidly than Parkinson’s and present with additional symptoms such as early falling, dementia or hallucinations. Moreover, unlike PD, parkinsonism may not respond to levodopa therapy or it may fail to respond to levodopa therapy long term [39].

The global prevalence of PD has more than doubled from 2·5 million people diagnosed in 1990 to 6·1 million people in 2016 [23], a worrying trend that looks set to continue with prevalence predicted to double again to 12 million diagnoses by 2050 [23]. In the U.K. around 137,000 people are currently living with PD, however due to population growth and an increasingly ageing population, it is estimated that prevalence of PD may be set to increase by 23.2% by 2025 [35].

Pain is experienced by the majority of PwP [8]. It is most often of nociceptive origin, but may also be ascribed to neuropathic (radicular or central) or miscellaneous sources. The validated King's Parkinson's Disease Pain Scale describes seven domains where pain can be salient in PD: musculoskeletal pain, chronic body pain (central or visceral), fluctuation-related pain, nocturnal pain, oro-facial pain, pain with discolouration/oedema/swelling, and radicular pain. The most frequently observed pain in PD is associated with musculoskeletal pain. Pain in PD has also been found to be associated with rigidity, a decrease in daily living activity, depression, and a reduction in overall quality of life (QoL). Chronic pain in PD has been found to represent the most important factor affecting health related QoL [37].

Support for pain management is limited in Parkinson’s [5, 11, 21, 27]. Between 50% [5] and 63% [27] of PwP reporting pain have not received pharmacological or non-pharmacological management. Crucially, a gap in knowledge exists concerning pain self-management and behavioural interventions for PwP. Due to the long-term nature of Parkinson’s, there is an urgent need to understand the skills and behaviours PwP require to facilitate active involvement in pain management [51]. Current reviews provide scant information concerning behaviour change interventions for self-management for PwP or specificity regarding how PwP can be supported to proactively manage pain.

A systematic review of randomised controlled trials (RCTs) for pharmacological and non-pharmacological therapies for pain and Parkinson’s indicates that further evaluative research is needed, specifically with reference to patient important outcomes [46]. The review by Qureshi et al. [46] demonstrated some promise for pharmacological approaches to pain management, however research was limited for non-pharmacological approaches. Non-pharmacological options should be considered for PwP [1, 25, 33], particularly for older people due to the increased risks with polypharmacy and co-morbidity [19]. A systematic review of non-pharmacological interventions for pain for PwP identified intervention studies including exercise, hydrotherapy, massage, acupuncture and neuromodulation [47]. Whilst a range of quantitative designs were eligible for inclusion, search terms were restricted to these specific interventions and did not consider behavioural interventions and self-management.

Guidelines for pain management in the general population advocate self-management [19, 34], education focusing on pain [19, 45], reduction of maladaptive attitudes/beliefs [1], individualised exercise [1, 34, 47] and signposting to self-help resources [34].

However, behavioural and self-management interventions are seldom included in systematic reviews [46, 47]. Self-management is discussed in a systematic review and meta-analysis reporting on clinical effectiveness of self-management interventions in Parkinson’s disease [44]. However, interventions that were focused specifically on pain self-management for PwP were not elucidated. This suggests that a broader scope of both the published and grey literature is required to understand pain management in Parkinson’s.

Moreover, there is evidence to suggest that PwP would value support to self-manage pain that was not based on pharmacological intervention alone [33]. For example, a recent study evaluating self-management support for pain and comorbidities using cognitive behavioural principles to support behaviour change demonstrated some promise, but was limited to a small sample of participants with a neurological condition [31]. Whilst there is no gold standard definition of self-management, it encompasses an individual’s ability to manage the physical, psychological and social impact of living with a health condition [4, 51, 53] with support if required [9, 51, 53]. Therefore, theory-informed behavioural interventions should be considered in relation to PwP which support active involvement in pain self-management. Of further interest is how behaviour change involves co-ordinated activities to change specific behavioural patterns [30], especially how behaviour change can support self-management of pain. This broad understanding of self-management provides potential for a wide range of different resources, interventions and professional involvement to be explored, as well as a broad spectrum of study designs to capture this.

The COM-B (Capability-Opportunity-Motivation-Behaviour Change) model [28–30] and Theoretical Domains Framework (TDF) [12] will provide a theoretical framework for synthesising evidence in this review. The COM-B model helps to identify how capability, opportunity and motivation interact to produce behaviour [52]. The TDF offers a comprehensive, theory-informed approach to identify determinants of behaviour [3]. It comprises 14 domains that represent a synthesis of 33 behavioural change theories [22, 29] and although it is a theoretical framework and not a theory, it provides a theoretical lens through which to identify cognitive, affective, social and environmental influences on behaviour. This will facilitate identification of specific barriers and enablers to pain management and behavioural determinants that support self-management for the purpose of this review. This is important to inform future interventions for the management of pain in Parkinson’s. The COM-B works well in conjunction with the TDF. Together, the COM-B model and TDF framework will support the identification of those behaviour change components that may support self-management of pain in PwP. Used in conjunction, the COM-B model and TDF framework offer a joint model that may yield a more granular understanding of the processes associated with behaviour change [3]. To our knowledge, this is the first review on pain management in Parkinson’s that takes advantage of the capabilities of both the COM-B model and TDF.

This review will adopt a biopsychosocial approach to explore what is available for PwP to support pain management, including how behaviour change interventions, education and pain self-management strategies might support this. A biopsychosocial perspective can include physical functioning, emotional reactivity and cognition to address all the key domains affected by chronic pain [7]. Recent research reveals potential disparities regarding how pain is managed by PwP from different ethnic backgrounds with respect to analgesia [48]. This review aims to explore and extract any relevant information regarding this in relation to wider pain management.

Overall, there is a need to explore the evidence base in terms of what has been done, focusing on self-management of pain for PwP, what skills and strategies are available to support PwP and whether they can be explained with reference to theory to inform future intervention development and evaluation. This review forms part of an ongoing, broader study of pain in Parkinson’s which builds on the largest ever pain in Parkinson’s study originally conducted by Silverdale et al. [50].

### Aims and objectives

A scoping review will be undertaken to examine and map the available evidence which has been produced in relation to pain management in Parkinson’s, with a focus on behavioural interventions, resources and/or professionals to support PwPs' self-management of pain. The purpose of this review is to synthesise the findings and present an overview of the evidence that summarises the range of interventions relating to the management of pain available to PwP. This summary will:

1. describe what is available for PwP who are living with pain to support self-management.
2. identify the characteristics of support for pain self-management.
3. identify the content of self-management interventions or resources for pain in PwP.
4. identify how these interventions or resources have been evaluated (if applicable).
5. identify what behaviour change components have been used in pain management support interventions or resources for PwP (this will be assisted by the COM-B model and TDF).
6. identify barriers and enablers to pain management in Parkinson’s and map these to the TDF and COM-B.
7. explore gaps in current provision of pain management.

## Methods and analysis

The scoping review will adhere to Arksey and O’Malley’s [2] methodological framework, including enhancements by Levac et al. [26], Peters et al. [40, 42, 43] and the Joanna Briggs Institute (JBI) [41]. In accordance with Levac et al.’s [26] recommendations, the review protocol and findings/analysis will be shared with key stakeholders via an expert panel consisting of clinicians and academics who are experts in the field of Parkinson’s, as well as with a lay panel of representatives who are either living with Parkinson’s or acting as a carer for someone with Parkinson’s. This will provide valuable feedback to assist in guiding the review, as well as helping to inform the significance of the various findings and any insights that can be gleaned, including the pinpointing of any gaps in current provision. Panel members will be invited because of their ability to contribute valuable insights to the review, including how it might be conducted and the key foci to address, based on their clinical/academic and/or lived experience of Parkinson’s.

This review will follow recommendations from the PRISMA-ScR guidelines for the reporting of scoping reviews. Details of interventions (when relevant) will be captured using the TIDieR Tool, i.e., brief name, rationale/theory/goal of the intervention, any physical or informational materials used in the intervention, procedures, interventionalist, how, when, where, how much, whether tailored/personalised, modifications, and any outcomes recorded.

This scoping review will include literature generated from 2010 since only a small amount of research exists that focuses specifically on PwPs' views on pain [33]. As recently as 2015 pain was still seen to represent an unmet need in PD [13].

It was also deemed appropriate to draw upon the most contemporary evidence, particularly since it is only in the last few years that there been increasing interest in the use of self-management approaches for features of Parkinson’s disease [44] and a primary objective of this review is to examine and map behavioural interventions, resources and/or how professionals support PwPs' self-management of pain. However, if no date is included on any relevant or insightful grey literature, this will be still be included to avoid overlooking key documents.

Consistent with JBI [40, 41], *p.19*) guidelines this scoping review will include a review of the grey literature. Grey literature can increase the comprehensiveness and timeliness of reviews and generate a more balanced picture of available evidence [38]. The dearth of published research on pain management in Parkinson’s makes the inclusion of grey literature especially important in this review, particularly in view of the fact that guidance on pain management in Parkinson’s tends to be available from unpublished sources, e.g., prominent.org Websites and in particular: Parkinson’s UK, Parkinson's Europe (parkinsonseurope.org), Davis Phinney Foundation, michaeljfox.org, Parkinson's Foundation, Canadian Parkinson’s Disease society, Live well with Parkinson's and Cure Parkinson’s Trust. These.org Websites will be searched in a bid to find PDF. reports that inform pain management in Parkinson’s. (A fuller list of identified sources is outlined in the next section). Selective focus on prominent Parkinson’s Websites should help increase the likelihood of retrieving grey literature that is relevant to the review’s focus while overcoming the difficulty inherent in Internet searches for specific grey literature that these can become unwieldy, unpredictable and time consuming to execute [6]. Selective focus will also be employed to mitigate the inherent difficulty in ensuring high-quality information from health Websites [36] and to help ensure grey literature will be extracted from reputable sources. In addition, the focus on locating PDFs within selected Websites will help to overcome the challenge of ensuring the completeness and accuracy of information [20, 32].

The following six stages based on Arksey and O’Malley’s methodological framework will be completed

### Stage 1: Identify the research questions

The proposed scoping review aims to establish a comprehensive understanding of the interventions and resources available to support behavioural interventions and pain self-management for PwP. Consistent with the operational definition of ‘PwP’ adopted for this review, the scoping review will be focused on people with a diagnosis of Parkinson’s disease as according to the UK Brain Bank Criteria. The review is not aimed towards people who demonstrate features indicating another type of degenerative parkinsonism, e.g., progressive supranuclear palsy.

The research questions seek to address the following:

1. explore what is available to support PwP living with pain/what practices are available.
2. identify the key characteristics of support for pain management available.
3. identify the barriers and enablers to pain self-management.
4. pinpoint of any gaps in current provision.

### Stage 2: Identify relevant studies

An initial search of databases was undertaken to establish a list of relevant search terms (*see detailed ‘Search strategy’ section below*) combined with the Boolean operator ‘AND’ within the selection of ‘English language.’

### Search strategy

A four-step search strategy will be utilised.

The first step involved an initial limited search of PubMed Central (PMC), APA PsycINFO (EBSCO) and grey literature sources using Advanced Google Search (limited to.org websites and.pdf files in a bid to find reports from well-being or healthcare organisations). These databases/sources were selected for their comprehensiveness, as well as their capacity to identify evidence from a broad range of biological, psychosocial and social perspectives. For initial, iterative search terms the Medical Subject Headings (MeSH) database was consulted to identify the concepts and choose all appropriate terms, both descriptors and synonyms. Consistent with previous findings [49], a trial run using a combination of free text and MeSH headings search terms (Table 1) was found to be optimal in retrieving valid and appropriate search results which met the aims and objectives of this scoping review.

**Table 1:** Trial run using a combination of free text and MeSH headings search terms

Initial, iterative search terms (1^st^ step)
Parkinson’s disease AND Pain AND (1) behavio* (2) self-care (3) self-manag* (4) self-efficacy (5) coping (6) multidisciplinary (7) education (8) physical activity (9) exercise (10) goal setting (11) self-monitoring (12) problem solving (13) cognitive behavioural (14) motivational interviewing (15) Physiotherapy (16) allied health (17) occupational therapy (18) psychology (19) biopsychosocial (20) wellbeing (21) relaxation (22) pacing (23) social support (24) rehabilitation (25) health promotion (26) views (27) experiences (28) perceptions

The second step will include a hand search and analysis of the key text words contained in the title and abstract of retrieved papers/grey literature sources, and of the index terms used to describe the articles.

The third step will include a second separate search using all identified keywords/index terms across all included databases (Table 2).

**Table 2:** Third step including a second separate search using all identified keywords/index terms across all included databases

**Database**	**Search Terms employed (** ***3*** ***rd*** *** step*** **)**
PubMed (PMC)	
SCOPUS (Elsevier) (abstract and citation database)	
Web of Science	
APA PsycINFO (EBSCO)	
Applied Social Sciences Index & Abstracts (ASSIA)	
WorldCat	

The reference list of all identified reports/articles/grey literature sources will be hand searched for additional studies that meet the inclusion criteria.

The fourth step will be to follow this up with a more comprehensive, structured database search of records to examine pain management approaches from January, 2010 to present. This more comprehensive search will include the following databases, consistent with the broad biopsychosocial lens through which the review will be viewed:

- PubMed Central (PMC) (free full-text archive of biomedical and life sciences journal literature)
- Scopus (Elsevier) (abstract and citation database) (+ option to search books)
- Web of Science
- APA PsycINFO (EBSCO) (Psychology including focus on pain behaviours, beliefs, cognitions)
- Applied Social Sciences Index & Abstracts (ASSIA) (providing a social/sociological perspective)
- CINAHL
- MEDLINE

Grey literature searches:

- Prominent and reputable.org Websites and in particular: Parkinson’s UK, Parkinson's Europe (parkinsonseurope.org), Davis Phinney Foundation, michaeljfox.org, Parkinson's Foundation, Canadian Parkinson’s Disease society, Live well with Parkinson's and Cure Parkinson’s Trust.

The grey literature search aims to be comprehensive to avoid potentially missing important sources and will include the following additional searches:

- WorldCat
- Advanced Google Search in a bid to find PDF. reports that inform pain management in Parkinson’s limited to the following prominent.org websites: Parkinson’s UK, Parkinson's Europe (parkinsonseurope.org), Davis Phinney Foundation, michaeljfox.org, Parkinson's Foundation, Canadian Parkinson’s Disease society, Live well with Parkinson's and Cure Parkinson’s Trust
- Patient leaflets
- Clinical guidelines: APTA
- European Physiotherapy guidelines
- Parkinson's Foundation information leaflets, Parkinson's UK information leaflets
- Progress magazine – research magazine of Parkinson’s UK
- First start programme
- NHS resources (patient advice/information leaflets, Websites, etc.).

Two reviewers will independently complete study selection following inclusion criteria (*see Stage 3 below for detailed summary*) reflecting: (i) population (people living with Parkinson’s) (ii) concept (pain management) and (iii) context (any clinical setting, but also data contained under the category ‘grey literature’) and will chart the data. Reference duplicates between the databases will be identified and removed. Reasons for study exclusion will be recorded. Any disagreements/discrepancies that emerge regarding inclusion will be resolved by consensus or mediated by a third reviewer.

### Stage 3: Study selection (iterative application of post hoc inclusion/exclusion criteria)

#### Inclusion criteria

(i) Population
  This scoping review will consider published studies and grey literature that either include individuals with a diagnosis of Parkinson’s or resources that are targeted specifically towards PwP.
(ii) Concept
  Research is required to understand the approaches available for pain self-management in Parkinson’s to support individuals to be active in their treatment and to develop skills and behaviours to self-manage their pain [51]. While pharmacological approaches are of importance [24], so too are approaches that require more active participation and agency by individuals that may also empower individuals towards adaptive behaviour change [16].
  It is now increasingly recognised how individuals’ perception of pain can be mediated by factors such as cognitions, beliefs, sociocultural variables, learning, and emotional reactivity [17]. In particular, the capacity of individuals to adaptively change how they think, understand, react and ultimately behave towards pain may be instrumental to pain management. Changing behaviour requires an understanding of the influences (i.e., facilitators of and barriers to change) of behaviour in the context in which they occur [3] and arguably, facilitators and barriers also include non-physiological factors [16] and behavioural determinants and mechanisms of action that facilitate adaptive behaviour change in relation to how pain is perceived in Parkinson’s over the disease course.
(iii) Context
  This review will include quantitative and qualitative studies, as well as mixed methods studies in any setting. The review will also include grey literature such as patient leaflets and NHS Website advice to patients.
(iv) Sources
  This scoping review will consider any geographical area/setting/source, provided the extracted data meets the inclusion criteria and addresses the aims/objectives of the review. Language will be restricted to English. No limits will be imposed relating to study design.

#### Exclusion criteria

Literature/sources will be *excluded* if they meet the following criteria:

- they do not relate specifically to people with Parkinson’s
- they do not relate to skills and behaviours to support active involvement in pain management for PwP and/or approaches/strategies/interventions or advice concerning pain management
- articles/sources produced prior to 2010
- articles/sources narrowly focused on pharmacological interventions used either in isolation or in conjunction with other, similar pharmacological interventions, rather than included as part of a programme to encourage active participation in management of pain
- articles/sources that relate to pharmacological interventions which are still being trialled
- interventions that are not framed in the context of supporting individuals to be proactive in their treatment
- articles not written within English Language (due to the limited resources available for the review).

### Stage 4: Charting the data

#### Extraction of the results

Two independent reviewers will extract the data from included studies. Inclusion will be based on the inclusion criteria: (i) population (ii) concept (iii) context (iv) sources (*as detailed in Stage 3 above*). Data extraction will be achieved by charting the results to provide a logical/descriptive summary detailing what available evidence has been produced in relation to pain management in Parkinson’s. Key information to be charted is outlined in Table 3).

#### Article selection process

For this scoping review, the Preferred Reporting Items for Systematic Reviews and Meta-Analyses (PRISMA) flow diagram will be employed to document the search and visually represent all the studies identified in each step of the study selection process.

### Stage 5: Collating, summarising & reporting the results (Thematic analysis of the data & numerical analysis of the extent & nature of the studies (using Tables/Charts)

#### Presentation of the results

The findings will be presented in a tabular form as a map of the data extracted, aligned with the objectives and scope of the review. This table will also include a quantitative summary of the data extracted (descriptive statistics), alongside chronicling the characteristics of support for pain management available, orientation of the approach employed (e.g., biological, psychological or social or a combination of these), any identifiable TDF domains and COM-B components and any behaviours and outcomes (i.e., of an intervention) where this is reported.

Due to the omission of any discussion of behavioural change/behavioural change theory in the most recent pain management and Parkinson’s reviews [44, 46, 47] use of the COM-B model and TDF will be applied retrospectively to the interventions identified and/or views expressed or resources enumerated, where behavioural change is not already made explicit.

In addition, Thematic analysis (TA) will be used to analyse patterns in the data and TA has been selected for its proficiency in organising, analysing, and reporting patterns to generate themes and sub-themes within data in rich detail [15]. The six-phase guide to conducting TA, as outlined by Braun and Clarke [10, 14], will be employed:

(i) Familiarisation with the data.
(ii) Generating initial codes.
(iii) Organisation of the initial codes into patterns to generate themes.
(iv) Reviewing themes (checking themes against raw data to ensure a good fit and reclassification of themes into levels).
(v) Defining and naming themes.
(vi) Interpretation.

NVivo software will be used for data management and all data will be coded and organised using this software as a helpful tool for sorting, organising, and analysing the data [18]. Theme development will be a collaborative process involving all three core researchers (MP, KB and JN) regularly meeting together to discuss themes and sub-themes as these are revealed within NVivo.

### Stage 6: Consultation

An iterative approach will be adopted in which the findings/analysis detailing key themes and sub-themes will be presented as a summary brief (sent out in advance of meetings to be held via Microsoft Teams) to an expert panel consisting of clinicians and/or academics who are expert in the field of Parkinson’s. Their critical feedback will be key as the range of backgrounds from collaborators (Psychology (× 2), Parkinson’s Nurse (× 1), Physiotherapy (× 5), Clinical rehabilitation (× 1), Consultant Neurologist (× 1) will ensure multiple perspectives and specialisms to inform data analysis. There will be several meetings among the wider research team (collaborators) for peer debrief to discuss the themes and sub-themes and their implications.

A summary brief will also be sent out at around the same time to the study PPI group. The PPI group will comprise a separate lay panel of representatives who are either living with Parkinson’s (*n* = 3) or acting as a long-term carer for someone with Parkinson’s (*n* = 1) who will be similarly consulted on the developing themes and sub-themes and how this informs the aims/objectives of the review. The lay panel will be reimbursed for their valuable time following each meeting via central University funding for PPI.

Expert and lay panel feedback will help to inform the significance of the various findings and highlight any additional insights to be gleaned from the review in relation to pain management in Parkinson’s.

A second meeting with the expert and lay panels will provide an opportunity to present any revised reports that may have been adjusted in light of the panels' initial feedback. A final summary report will be produced to illustrate Stakeholder input and how this influenced the review. Subsequent draft manuscripts presenting the key findings from the review will also be discussed with the expert and lay panel for critical review prior to their submission to a peer-reviewed journal.

## Discussion

### What this review will add to existing knowledge

This review seeks to address the current gap in knowledge regarding the support that is specifically available to help PwP to self-manage pain over the full disease trajectory. In particular, the bespoke skills and behaviours PwP require to support their active involvement in pain self-management. To our knowledge this is the first time such a review will be assisted by the COM-B model and TDF that will in turn help to identify specific behaviour change components that may support self-management of pain in PwP. To the authors’ knowledge this is the first review on pain management in Parkinson’s that takes advantage of the capabilities of both the TDF and COM-B model.

### Dissemination

The findings of this scoping review will be presented at a conference and prepared as a manuscript for publication. Furthermore, findings will facilitate understanding about what is available and gaps in provision and to inform the development of a pain management intervention for PwP. Working with the expert panel and patient advisory group will support further dissemination to both professionals and PwP.

## Data Availability

Not applicable.
